# Supplementary material for: Directional Changes in the Intestinal Bacterial Community in Black Soldier Fly (Hermetia illucens) Larvae
Source: Animals (Basel). 2021 Dec 6;11(12):3475. doi: 10.3390/ani11123475 (PMC8697953; doi:10.3390/ani11123475)
Supplement: Supplementary file 1 [file animals-11-03475-s001.zip › animals-1438858-supplementary.pdf]

Table S1. Composition of bacteria in seven different groups at phylum level (%).

Phylum	A	B	C	D	E	F	G
Proteobacteria	83.8	82.66	76.54	85.41	77.7	80.99	84.63
Firmicutes	14	16.75	22.41	14.23	20.3	16.53	14.27
Bacteroidetes	0.66	0.18	0.89	0.19	1.8	2.33	0.93
Other	1.54	0.41	0.16	0.17	0.2	0.15	0.17

The letter A refers to the control. The letters B, C, D, E, F, and G refer to the larvae intestinal bacteria fed with sterile FW that was inoculated with *Lysinibacillus sphaericus*, *Proteus mirabilis*, *Citrobacter freundii*, *Pseudocitrobacter faecalis*, *Pseudocitrobacter anthropi*, *and Enterococcus faecalis*, respectively.

Table S2. Composition of bacteria in seven different groups at genus level (%).

Name	A	B	C	D	E	F	G
Ignatzschineria	49.69	42.62	40.8	54.83	46.39	31.26	49.76
Providencia	14.93	16.08	17.15	17.74	16.18	20.11	22.08
Proteus	9.2	12.3	8.38	4.64	5.26	16.69	4.56
Klebsiella	5.02	3.59	4.78	3.56	3.65	6.73	3.39
Vagococcus	3.08	6.9	6.71	1.4	4.04	5.34	1.77
Morganella	2.48	5.01	4.13	2.06	4.14	2.88	3.57
Enterococcus	2.6	2	4.22	7.58	2.13	2.26	2.81
Tissierella	1.96	1.02	2.1	1.2	4.85	1.32	2.13
Erysipelothrix	1.61	1.25	2.77	1.24	3.02	0.87	2.08
Romboutsia	1.94	1.44	3.44	0.19	0.39	1.1	1.21
Bacteroides	0.26	0.11	0.8	0.12	1.05	2.09	0.89
Terrisporobacter	0.2	0.07	0.71	0.11	0.25	2.48	0.66
Bacillus	0.39	1.09	0.55	0.18	0.14	0.95	0.21
Paenalcaligenes	0.03	1.63	0.04	0.59	0.03	0.67	0.01
Peptostreptococcus	0.27	0.17	0.08	0.07	1.04	0.25	0.28
Unclassified *Enterococcaceae*	0.62	1.61	0.84	1.62	0.24	0.64	1.22
Unclassified *Clostridiales_ Incertae_Sedis_XI*	0.5	0.19	0.7	0.19	2.76	0.5	1.03
Unclassified *Enterobacteriaceae*	0.51	0.49	0.4	1.14	0.56	1.46	0.3
Other	4.7	2.43	1.4	1.54	3.9	2.41	2.06

The letter A refers to the control. The letters B, C, D, E, F, and G refer to the larvae intestinal bacteria fed with sterile FW that was inoculated with *Lysinibacillus sphaericus*, *Proteus mirabilis*, *Citrobacter freundii*, *Pseudocitrobacter faecalis*, *Pseudocitrobacter anthropi*, *and Enterococcus faecalis*, respectively.

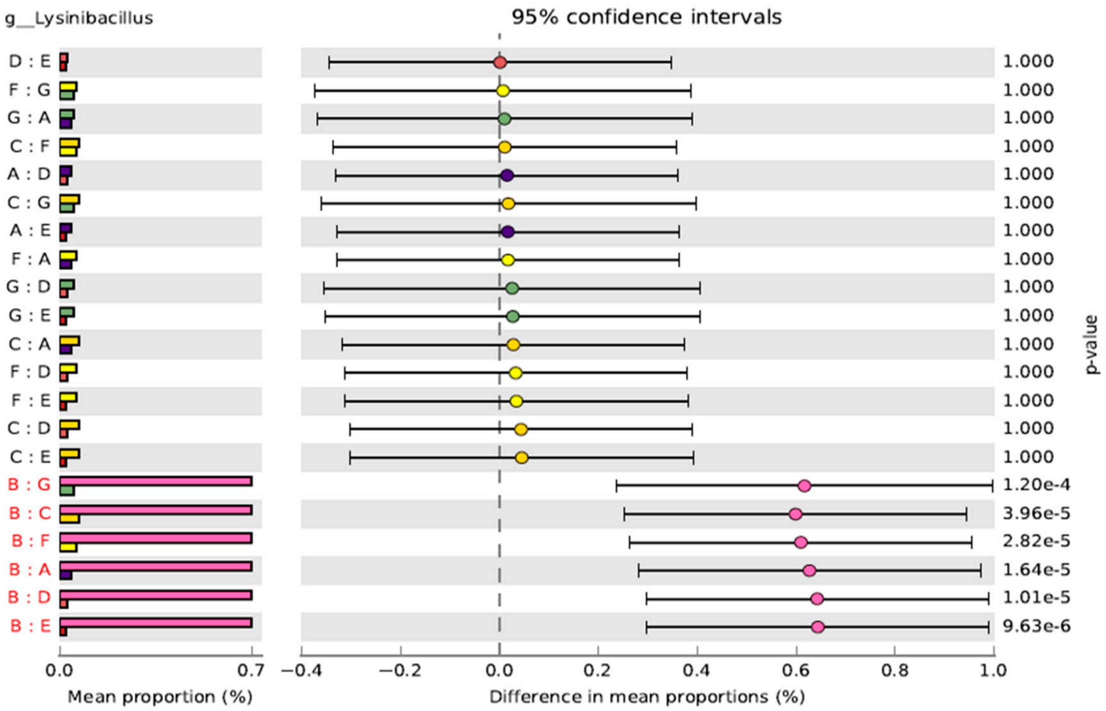

Figure S1. *Post hoc* analysis results of *Lysinibacillus* in all groups. The left of the picture shows the abundance ratios of *Lysinibacillus* in the different groups. Different colors represent different groups. The middle shows the ratio of differences between groups within the 95% confidence interval. The comparison between the groups marked in red denotes significant differences ($P < 0.05$), as obtained by pairwise *post hoc* tests.
